# Effects of the integrated Community Case Management of Childhood Illness Strategy on Child Mortality in Ethiopia: A Cluster Randomized Trial

**DOI:** 10.4269/ajtmh.15-0586

**Published:** 2016-03-02

**Authors:** Agbessi Amouzou, Elizabeth Hazel, Bryan Shaw, Nathan P. Miller, Mengistu Tafesse, Yared Mekonnen, Lawrence H. Moulton, Jennifer Bryce, Robert E. Black

**Affiliations:** Institute for International Programs, Department of International Health, Johns Hopkins Bloomberg School of Public Health, Baltimore, Maryland; Alliance for Better Health Services Private Limited Company, Addis Ababa, Ethiopia; Mela Research Private Limited Company, Addis Ababa, Ethiopia

## Abstract

We conducted a cluster randomized trial of the effects of the integrated community case management of childhood illness (iCCM) strategy on careseeking for and coverage of correct treatment of suspected pneumonia, diarrhea, and malaria, and mortality among children aged 2–59 months in 31 districts of the Oromia region of Ethiopia. We conducted baseline and endline coverage and mortality surveys approximately 2 years apart, and assessed program strength after about 1 year of implementation. Results showed strong iCCM implementation, with iCCM-trained workers providing generally good quality of care. However, few sick children were taken to iCCM providers (average 16 per month). Difference in differences analyses revealed that careseeking for childhood illness was low and similar in both study arms at baseline and endline, and increased only marginally in intervention (22.9–25.7%) and comparison (23.3–29.3%) areas over the study period (*P* = 0.77). Mortality declined at similar rates in both study arms. Ethiopia's iCCM program did not generate levels of demand and utilization sufficient to achieve significant increases in intervention coverage and a resulting acceleration in reductions in child mortality. This evaluation has allowed Ethiopia to strengthen its strategic approaches to increasing population demand and use of iCCM services.

## Introduction

Progress in child survival has been too slow in most low-income countries to achieve the fourth Millennium Development Goal (MDG4) of reducing mortality among children under five by two-thirds between 1990 and 2015.^1^,^2^ Over six million children died in 2013 from preventable causes, largely in low-income countries.^2^ In sub-Saharan Africa, for example, where half of worldwide deaths occurred, neonatal disorders caused a third of these deaths, and pneumonia, diarrhea, and malaria were responsible for 40%.^3^ Undernutrition is associated with nearly half of the deaths of children under five through its synergistic relationship with infectious diseases.^4^ However, low-cost and proven high-impact preventive and curative interventions exist to reduce neonatal and child deaths rapidly, and can be scaled up to reach high coverage levels through effective delivery strategies. Inequities by household wealth and distance in access to curative care services and in rates of child mortality remain large, and strategies are needed to reach the poorest and most remote segments of the population.^1^,^5^–^8^

Many countries have adopted a strategy of integrated community case management (iCCM) to tackle the slow and inequitable progress in child survival. iCCM programs train and support community health workers (CHWs) to assess and treat the three major infectious causes of death among children under five: uncomplicated cases of pneumonia with antibiotics, malaria with artemisinin-based combination therapy (ACT), and diarrhea with oral rehydration salts (ORS) and zinc. In some settings, the iCCM approach also includes detection and community-based management of severe acute malnutrition with ready-to-use therapeutic foods (RUTF). Cases of severe disease seen by these workers are referred to higher level facilities, after some pre-referral treatment. By bringing services into communities, closer to where people live, the iCCM strategy is also expected to reduce inequities in access to prompt care and treatment for these life-threatening childhood illnesses.^9^,^10^

There is a growing number of studies demonstrating that CHWs are able to implement iCCM and the strategy is associated with improvements in the quality of child health care at community level, albeit with room for improvement for children with pneumonia and severe illness.^11^–^15^ However, the effectiveness of iCCM in accelerating reductions in mortality among children under five has not been demonstrated. Similar to the integrated management of childhood illnesses (IMCI), which has been rolled out in many low-income countries, the theoretical model underpinning iCCM is strong and appealing because the aim is to reach more children with proven, efficacious interventions. However, the multi-country evaluation of IMCI suggested that several programmatic, health system, and demand factors must be taken into account in the impact pathway.^16^–^18^ The generic iCCM impact model includes the following five steps, each one leading to the next: 1) implementation of a strong iCCM program with high-quality care characterized by appropriate training of CHWs, regular supervision and monitoring, and continuous supply of drugs and commodities; 2) accessibility of the program to a high proportion of the target population; 3) improved careseeking and service utilization; 4) increased coverage of proven interventions for sick child care; and 5) improved nutritional status and accelerated declines in mortality. All elements of this impact model must be present to observe measurable declines in child mortality. Thus, although many countries have scaled up iCCM, the need for a full evaluation of each step remains warranted to generate country and global learning and encourage further international and financial support for the strategy.

Ethiopia, the second most populous country in Africa, experiences high mortality rates in children under five, but is also one of the few countries that has achieved rapid declines in under-five mortality. The 2014 report of the United Nations Inter-Agency Group for Mortality Estimation estimated that under-five mortality decreased by 69%, from 205 deaths per 1,000 live births in 1990 to 64 in 2013, outpacing the MDG4 target of a two-thirds reduction.^2^ This trend was confirmed by direct measures of under-five mortality in the 2005 and 2011 demographic and health surveys.^19^,^20^ Ethiopia made a commitment to achieve the MDG4 target in 2004, reflected in the launch of the national Health Extension Program (HEP). The HEP aims to provide health care to under-served populations, with an emphasis on community self-reliance and sustainability.^21^–^23^ The key strategy of the HEP program is to train and support over 30,000 health extension workers (HEWs) to provide and promote preventive and selected curative health-care services at the community level. Until 2009, HEWs were trained to treat diarrhea with ORS, malaria with ACT after a positive rapid diagnostic test, and severe acute malnutrition with RUTF. Pneumonia cases were referred to the nearest health center. An estimated 22% of under-five deaths in Ethiopia were attributable to pneumonia in 2006,^24^ and in 2009, the government agreed to expand the iCCM strategy to include management of uncomplicated pneumonia with antibiotics by HEWs. Besides clinical services, HEWs are also responsible for educating their community on preventive behaviors focusing on 16 key messages. They work generally with community volunteers during community health education and identification of vital events such as pregnancies, births, and deaths.

The initial rollout of the iCCM program was supported by the Catalytic Initiative to Save a Million Lives, with financial support from the Department of Foreign Affairs, Trade and Development Canada through the United Nations Children's Fund (UNICEF). iCCM implementation began in the four biggest regions of the country (Amhara; Oromia; Southern Nations, Nationalities, and Peoples; and Tigray) and later expanded to other regions. The rollout was rapid and covered entire regions, except Oromia, where it was phased in by zone or *woreda* (district).

We report here an evaluation of the iCCM strategy as implemented in Oromia region, Ethiopia, which assessed each step in the impact model outlined above. Steps 1) and 2) have been reported elsewhere, as have the results of a qualitative study investigating step 3).^14^,^25^ Here we provide the first report of steps 4) and 5), and interpret the overall findings.

## Methods

The evaluation used a cluster randomized trial design and was registered at clinicaltrials.gov under the number NCT01606267. Ethical clearance was obtained from the Johns Hopkins Bloomberg School of Public Health Institutional Review Board, the Ethiopian Public Health Association, and the Oromia Regional Health Bureau. Fieldworkers read the informed consent statement to each participant in their local language and obtained oral consent before conducting the interview.

### Study site.

In collaboration with the Ministry of Health and UNICEF-Ethiopia, we selected Oromia region for the evaluation. The national iCCM implementation plan called for phased introduction of iCCM in Oromia region, due to its large geographic size (the largest region in the country, with over 30 million people or about 36% of the national population). This phasing provided an opportunity to randomize woredas for immediate implementation (“phase 1” or intervention), and others for later implementation (“phase 2” or comparison). This allowed for a rigorous design to generate unequivocal results on program impact.

**Figure 1.**
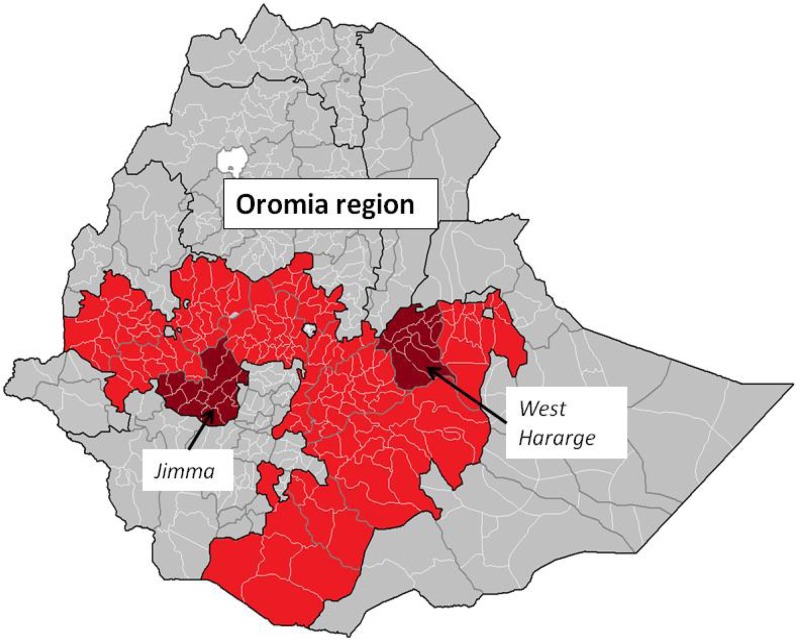
Map of evaluation zones in Oromia region, Ethiopia.

Within the Oromia region, Jimma and West Hararghe zones were selected for the evaluation in discussion with the Oromia Regional Health Bureau and iCCM implementing partners. These two zones were selected because of their large population size and number of woredas (to maximize sample size of randomized units), geographic representation (Jimma is in western Oromia while West Hararghe is in the eastern part of the region, and they are about equidistant from the capital city, Addis Ababa), and the reported strength of the implementing partner (to ensure that the evaluation was conducted within the agreed timeline, it was critical that implementation be scaled up rapidly and strongly). The Last Ten Kilometers project is an implementing partner, already present in Jimma, covering most woredas with maternal, newborn, and child health activities. The Integrated Family Health Program was another strong implementing partner present in West Hararghe. Both partners had already been contracted by the Oromia Regional Health Bureau to lead the iCCM implementation in these two zones with support from UNICEF. Figure 1

shows the map of Ethiopia with Oromia region and the two zones indicated.

### Intervention.

The intervention consisted of the iCCM program as implemented in the selected intervention areas. The comparison areas continued to implement routine HEP, which includes community case management of malaria, diarrhea, and severe acute malnutrition. A description of the Ethiopian iCCM scale-up is presented elsewhere.^26^ The iCCM program relies on HEWs, who were recruited at the national level and underwent 1-year training as part of the HEP. They are considered government employees with salary and operate from fixed health posts within a *kebele* or sub-district, although they are expected to spend a significant portion of their time in their community, providing house-to-house clinical, education, and preventive services. There are generally two HEWs covering a kebele of about 5,000 people. HEWs were deployed in rural kebeles throughout the evaluation zones. This model of iCCM is different from other forms that rely on lay volunteers, appointed by communities themselves, and covering generally smaller catchment areas.

Although the only clinical difference between the intervention and comparison areas was the addition of community case management of pneumonia, intervention areas received an enhanced iCCM package that included supplemental 6-day training of HEWs, a temporary system for provision of drugs and commodities, new registers, increased supportive supervision, and biannual performance reviews and clinical mentoring. Supplemental Web Annex, Table 1 shows differences between the iCCM program and routine HEP in terms of program inputs and case management.

### Study design.

The independent evaluation used a cluster randomized design with clusters represented by woredas. All 31 rural woredas in the two evaluation zones were randomized to intervention (16 woredas) and comparison (15 woredas) areas using restricted randomization.^27^ The randomization was stratified by zone and executed to ensure balance to within a relative 10% for the woreda population, the ratio of health posts to population, the number of HEWs per population, and the percent of households trained as model families, and to within a relative 20% for the percent of kebeles (subdistricts) with malaria. Supplemental Web Annex, Figures 2 and 3 show the two selected zones with randomized intervention and comparison woredas. Supplemental Web Annex, Figure 1 presents a summary of the evaluation design. Results of the assessment of implementation strength and quality of care and the qualitative study are available elsewhere.^14^,^25^ Here, we focus on the primary outcomes of interest (careseeking, treatment, and mortality), although we draw on the full set of evaluation results in our interpretation of the findings.

### Data.

A household survey was conducted from December 25, 2010 to February 24, 2011 in 3,200 households to measure baseline coverage levels of child health interventions and nutritional status. This sample size was sufficient to measure treatment of suspected pneumonia with a precision of ± five percentage points. The survey sample was embedded within a larger mortality sample of 11,000 households being implemented at the same time, which used the 2007 population census enumeration areas (EAs) as a sampling frame. The sample of the larger mortality survey was based on a stratified two-stage cluster sampling with EAs representing the clusters and woredas used as strata. In the first stage, EAs were selected within each woreda using systematic sampling with probability proportionate to size in households. The list of households in each selected EA was updated through a complete listing of households. A sample of 25 households was then selected in each EA using systematic sampling with equal probability. EAs for the baseline coverage survey were selected from the EAs selected for the larger mortality survey using systematic sampling with equal probability. Four EAs were selected in each woreda for a sample of 100 households per woreda.

The implementation of the iCCM program in the randomized intervention woredas started immediately after the completion of the baseline survey. An implementation snapshot and quality of care study was conducted in June 2012, over 1 year after the start of the iCCM program in the evaluation areas, to assess the strength of iCCM implementation, utilization of the program, and quality of sick child care provided by HEWs.^14^ In December 2012, we conducted a qualitative study to assess barriers to use of health posts by the population as part of a proactive strategy to provide early feedback to program implementers on strategies to increase demand for iCCM services.^25^

Between February and May 2013, an endline survey was conducted in 28,000 households to measure coverage of child health interventions, nutritional status, and child mortality. Child mortality measurement was based on a full birth history interview administered to women aged 15–49 years. The sample was computed to detect at least 21% difference in reduction in mortality among children under five between intervention and comparison areas. Details of the sample size calculation are included in Supplemental Web Annex, Table 2. The survey protocol included a full birth history to measure child mortality, but coverage of child health interventions and nutritional status were measured in a nested sample of 12,000 households. Similar to the baseline survey, the samples used the 2007 population census sampling frame and a stratified two-stage cluster sampling, with each woreda representing a stratum. The Institute for International Programs at Johns Hopkins University, Baltimore, MD, collaborated with the local research firm Mela Research PLC, Addis Ababa, Ethiopia, to carry out the baseline survey, and with Alliance for Better Health Services PLC, Addis Ababa, Ethiopia, to conduct the implementation snapshot and quality of care study, the qualitative study, and the endline survey.

### Analysis.

Although woredas were randomized into intervention and comparison areas, we first assessed the equivalence of study groups based on selected demographic and socioeconomic characteristics of the sample. Child health coverage indicators included careseeking for childhood illnesses (pneumonia, diarrhea, and fever), careseeking for childhood illnesses within 24 hours of onset of illness, treatment of childhood illnesses with the appropriate drug (antibiotics for pneumonia, ORS for diarrhea, and ACT for fever), and careseeking for sick children who received appropriate drug treatment within 24 hours of onset of illness. For careseeking, data was collected on all places where care was sought. However, only a small percentage reported multiple providers.

We computed averages of coverage indicators by woreda and average of averages across woreda. We conducted a *t* test of the coverage level across woreda to determine the significance of differences in indicator levels between intervention and comparison woredas at baseline and endline. We computed difference in differences by fitting generalized linear mixed-effect models (with logit link function) of each coverage indicator with three levels (individual child, EA, and woreda). The interaction effect between the intervention dummy variable and the indicator variable for the baseline and endline provides the difference in differences in the effect of program. In each analysis, we report the *P* value of the interaction, indicating whether a significant difference in differences was found for the indicator being analyzed. Tests of within-arm changes over time are given for descriptive purposes only, as they are not controlled for secular trends. No adjustments have been made for multiple comparisons.

We analyzed mortality among children under five and aged 2–59 months. Children under the age of 2 months may be seen by HEWs, but the protocol at the time of the evaluation required that they all be referred to the nearest health center. Thus, the main target population of the iCCM program was children aged 2–59 months. Results of analysis based on all children under age five were also carried out and are presented in Supplemental Web Annex, Tables 3–7. The implementing partners reported that the scale-up of the iCCM program including training, equipping, and deploying the HEWs to start implementation was completed by July 2011. Thus, our period for mortality measurement was from August 2011 to January 2013 (18 months), during which time the implementation was sustained in intervention woredas, and the comparison woredas received only the routine HEP services. To minimize seasonality effects, a similar period (August 2009 to January 2011) was selected for computing baseline mortality. The full birth history module from the endline survey was used to measure the two mortality indicators retrospectively at baseline and endline. Average mortality rates across woredas were computed for each study arm and for the periods before and during the intervention. A difference in differences analysis was carried out to determine the impact of the iCCM program. Standard errors for the difference between baseline and endline in each group computed from a *t* test of the mortality rates across woreda were used to compute 95% confidence intervals for the difference in differences in the mortality between baseline and endline in the intervention and comparison areas. All analyses were conducted using STATA (College Station, TX) software version 13.0.^28^

## Results

Table 1 summarizes demographic and socioeconomic characteristics of the sampled households at baseline and endline and by study group. The distribution of the sampled household population by zone, the average household size, and the mean age of the head of the household are fairly similar between intervention and comparison areas at baseline and endline. Similarly, no substantial and systematic imbalance is noticeable between the intervention and comparison areas at baseline or endline for the number of eligible women interviewed in households, the number of eligible children, and the proportion of households headed by women. Slightly fewer households were headed by women in the intervention area than in the comparison area at baseline (13.2% and 16.7%, respectively), but the gap declined at endline to a similar proportion. Women's education level and the average ages of caretakers and children are also similar between intervention and comparison areas at baseline and endline.

### Careseeking for childhood illnesses.

Table 2 shows the proportion of children aged 2–59 months with illnesses in the 2 weeks preceding the survey for whom formal care was sought by type of illness. For children who had at least one illness (suspected pneumonia, diarrhea, or fever) in the past 2 weeks, there was no statistically significant increase in formal careseeking (from a government health facility, health post, or private clinic) in the intervention areas. In general, formal careseeking increased in both intervention and comparison areas, and if anything, slightly more so in comparison areas.

iCCM is delivered by HEWs based in health posts within kebeles. In general, one health post is present in each kebele, and there is one health center for every five kebeles. Thus, health posts are closer to the population and serve as extensions of first-level care within communities. Any effect of iCCM on careseeking would be observed by differentiating the care provider. Table 2 also shows the proportion of children aged 2–59 months who were reported to have had an illness in the past 2 weeks and for whom care was sought by type of illness and by type of provider, distinguishing HEWs, other public health facilities, private health facilities, and informal sources (shops, traditional healers, and friends).

Careseeking from HEWs remained generally low in the evaluation area. At endline, caretakers of slightly less than 10% of children aged 2–59 months with at least one of the three illnesses in the previous 2 weeks sought care from an HEW in intervention areas (9.3%) or comparison areas (7.7%). However, these proportions represent a statistically significant increase from the baseline level (5.1% in intervention and 3.6% in comparison areas). The increases in the proportions occurred equally in both study arms, making the difference in differences statistically insignificant. Similar patterns of increases were observed for all three illnesses.

Careseeking from other public health facilities (largely health centers) was higher than careseeking from HEWs. However, differences between baseline and endline in intervention areas were not statistically significant.

Decreases in levels of careseeking for childhood illnesses from private health facilities or informal sources were observed in both intervention and comparison groups, although the level of decrease was higher in intervention areas than that in comparison areas. There may be a shifting of careseeking from these sources to health posts or other public health facilities, but the shift appeared in both intervention and comparison areas.

### Treatment of pneumonia, diarrhea, and malaria.

Table 3 shows the percentage of children aged 2–59 months in intervention and comparison areas who had an illness in the 2 weeks preceding the baseline and endline surveys and were reported by their caregivers to have been treated with the recommended drug by type of illness. Overall, level of treatment with the recommended drug decreased between baseline and endline for each type of illness in the intervention areas, but remained unchanged in comparison areas, except for a significant improvement in the treatment of diarrhea.

By bringing care closer to homes, iCCM is expected to increase prompt careseeking for and treatment of illness within 24 hours of onset of the illness to avoid complications and death. Table 3 shows the percentage of children aged 2–59 months with suspected pneumonia or fever who received the recommended treatment drug within 24 hours of the illness onset. At endline, only 5.6% of children with suspected pneumonia received antibiotics promptly in intervention areas compared with 8.2% in comparison areas. These figures reflect a decrease from the measured level at baseline.

Table 3 also shows the percentage of sick children who received the recommended drug by type of treatment provider. Overall, coverage with correct treatment is very low for all types of providers, a reflection of the low level of careseeking for and treatment of the three illnesses.

The findings show that in general, receipt of correct treatment from other public health facilities increased slightly between baseline and endline in both study areas, but was statistically significant only for treatment of fever with ACT in comparison areas.

### Mortality.

Table 4 shows mortality rates for children aged 2–59 months. Overall, mortality among children aged 2–59 months decreased by 12.7% (from 49.0 to 42.8) in intervention areas, compared with 9.1% (from 45.0 to 40.9) in comparison areas. Neither decline is statistically significant, nor is the difference in differences.

## Discussion

The evaluation of the iCCM program as implemented in Jimma and West Haraghe zones of the Oromia region, using a cluster randomized design, showed that 18 months after full implementation, the program did not produce expected increases in coverage of careseeking for and appropriate treatment of suspected pneumonia, diarrhea, and fever, and consequently did not have an impact on mortality among children under five or children aged 2–59 months. The impact model outlined in the introduction provides a useful heuristic for examining the strengths and weaknesses of the program.

In terms of inputs and processes, the program appears to have been well designed and delivered as planned in the two evaluation zones. A program documentation exercise carried out in the two evaluation zones for the purpose of the evaluation found that between February and July 2011, the program was scaled up rapidly, using a 6-day training model and deploying 935 HEWs to cover 492 health posts. A total of 118 HEW supervisors identified from health centers were also trained in the two zones. The ratio of trainers to trainees was 1:5 and the training was skill-based, with clinical sessions comprising 58% of the total training time. During the training, HEWs received job aids such as child registration books, chart booklets displaying the iCCM clinical algorithm based on the IMCI algorithm, which were of high quality and in the local language. An exercise book walked the HEW through the various case management scenarios. There was an extensive daily supervision process during the training to identify participants who might be falling behind and support them to improve. During the training, a facilitator worked with small groups of five HEWs at the health center on the clinical practice sessions—including direct observation of sick child assessment for 1 day.

An assessment of the strength of iCCM implementation and quality of care provided by HEWs was carried out midway through the implementation period.^14^ The assessment showed that the program was strongly implemented, and HEWs provided fairly good quality of care. Nearly all HEWs surveyed were trained in iCCM, 87% had received supervision in the 3 months preceding the survey, and 85% had received supervision with clinical reinforcement. More than two-thirds of the health posts surveyed had all the essential iCCM commodities in stock on the day of the survey, and about half had experienced stock-outs lasting more than seven consecutive days of at least one essential commodity. In terms of quality of care, trained HEWs managed childhood illnesses correctly in about two-thirds of cases. However, only about a third of severe cases were managed correctly, and half of cases needing referral were actually referred. A key finding of the assessment was low utilization of the iCCM services. On average, HEWs saw only 16 sick children per month, a level that was estimated to be about 60–75% below what would be expected given the prevalence of common childhood illnesses.^14^ These results showing low utilization are consistent with a report from another recent study in other regions of the country that showed that cases of pneumonia, diarrhea, and malaria seen over an annual period were, respectively, only 8%, 1%, and 12% of the expected annual cases.^29^

Thus, although iCCM was rolled out comprehensively to health posts, and, therefore, increased population access to services, utilization of the services did not increase as expected. There are several factors likely to have contributed to this low utilization. First, the iCCM program, as delivered, put more emphasis on the supply side of health service delivery than on the demand side. Although the training of HEWs included orientation on demand-generating activities, it was left to the HEWs to inform community leaders, religious leaders, and other key informants about the availability of iCCM services, and to encourage parents to take sick children to the health post using the family health counseling booklet. No other nationwide demand generation strategies were undertaken to inform the population about the new and improved skills of the trained HEWs and the availability of integrated services at health posts. It is likely that the HEWs did not fully implement expected demand generation activities, especially given the many other demands on their time.

Second, most kebeles were geographically large and included many sparsely clustered villages with irregular topography, limiting careseeking at health posts. Likewise, HEWs did not have any means of transportation to facilitate their home visits and ensure that they covered their entire catchment areas to complete demand creation and health promotion activities.

Third, a qualitative study conducted from December 2012 to January 2013 in the evaluation areas to understand barriers to utilization of health posts revealed that acceptability of HEWs was often low due to a perceived lack of sensitivity, neglect of certain villages and populations, and perceived low-quality or inconvenient services at the health post. The perceived low quality of services provided by HEWs was reported to stem from experiences with the HEP before the intervention period, when the program did not include management of suspected pneumonia and experienced frequent stock-outs of drugs and unavailability of the HEWs at the health posts.^25^ In addition, although two HEWs are generally assigned to a health post as a strategy to maintain continued service provision at the post, the quality of care and qualitative studies showed that the HEWs are often absent from the health posts.

Fourth, the qualitative study found that social networks acted both to facilitate and hinder use of HEWs. Many mothers stated a preference for using the health post, but some were unable to do so due to objections or alternative careseeking preferences of gatekeepers, often mothers-in-law and husbands, and competing demands on their time.^25^

Finally, the implementation strength assessment revealed that only 12% of surveyed HEWs reported living in their catchment areas at least 1 year before their recruitment, training, and deployment as HEWs, suggesting that the majority of the HEWs were not recruited from the communities they were serving in. Their status as outsiders or relative newcomers may also have affected utilization.

We also assessed changes in other key interventions that could have differentially affected child health and mortality in the two studies (Supplemental Web Annex, Table 8). There were no significant differential changes in interventions such as use of insecticide-treated bednets by children under five, exclusive breast-feeding, and immunization (measles vaccine, three doses of pentavalent vaccine, and three doses of pneumococcal vaccine). Only vitamin A supplementation to children 6–59 months changed due to a significant increase in the comparison arm.

Given these challenges, it is not surprising that the iCCM program did not show any significant impact on either coverage or mortality within the first 2 years of its implementation in the Oromia region. Although delivered with reasonably high quality, the pathway to impact was weakened by the low utilization of the services by the population. The iCCM program, as planned and deployed in Ethiopia, did not include at the outset a strategy for generating demand and utilization of the services at health posts, which, as we showed, is a fatal flaw in the design and implementation of the program. The Ethiopian government has responded to these findings by launching a “Health Development Army” program with the aim of mobilizing up to three million community volunteers.^30^ It remains to be seen if this strategy will increase utilization of health posts, especially because high service quality must be continuously present at the health post level to achieve the needed increases in utilization. A recent study in three Ethiopian regions suggested that utilization of health posts by sick children aged 2–59 months appeared to have increased by 60% over the course of 1 year, although overall utilization remained low.^29^ Ethiopia can be congratulated on the progress made in rolling out iCCM thus far, and on providing an important opportunity for learning through this evaluation.

These results are specific to iCCM as implemented in Oromia region, and should be generalized with caution for three reasons. First, the “new” components of iCCM included only clinical management of pneumonia and strengthened support for implementation; differences relative to the comparison area can be expected to be higher in places where the counterfactual is no community-based curative services. Second, the period of full implementation assessed was only 18 months, perhaps too short to allow uptake of utilization to generate measurable effects at population level. Third, the model of iCCM implemented in Ethiopia rely on existing HEWs, who are government employees, recruited at national level, trained for 1 year, and deployed to the community. They operate from fixed health posts, covering large areas of about 5,000 people. Although they are expected to spend a significant percentage of their time in the community, it is unclear whether they actually do so and what sort of activities they perform. Our assessment of time spent the previous day found that on average, HEWs reported spending a total of 6 hours on work-related activities, of which 4 hours were spent at the health post, 0.5 hour spent providing clinical services in the community, and 0.9 hour spent on community education and preventive services. Although generally two HEWs are assigned to a health post, the unavailability of services in the community has been a barrier to the use of HEW services. The Ethiopian model of iCCM is different from other forms of iCCM, which rely on lay community volunteers, appointed by their own communities and covering relatively smaller catchment areas. With information generated by the evaluation and new efforts to increase demand, it should be possible to strengthen all steps and to further assess whether the program generates significant impact.

This first rigorous evaluation of the impact of the iCCM program at scale in Ethiopia provides a wealth of lessons that address critical steps needed for an effective iCCM program. Most importantly, it sheds light on the need to take into account community demand of services as an integral part of the iCCM strategy, even in settings where community health workers have been already deployed and working for years. This is a necessary factor on the pathway to impact and should not be simply an afterthought or an add-on but part of the conceptual framework for improved service utilization and coverage of careseeking. Careful pre-assessment of the epidemiological and service delivery context, including quality of care at health facility level, are essential in the design of the program. In addition, the results highlight the importance of strong evaluation designs, with counterfactuals—not only to provide impact results, but even more importantly to have an accurate understanding of the extent to which strategies and programs are performing as planned. Critical lessons can be learned for program improvement when evaluations include rigorous and timely assessment of impact.

## Supplementary Material

Supplemental Datas.

## Figures and Tables

**Table 1:** Selected demographic and socioeconomic characteristics of the samples at baseline and endline by study group

Characteristics	Baseline				Endline			
	Intervention		Comparison		Intervention		Comparison	
	Value	SD	Value	SD	Value	SD	Value	SD
Percent of household population by zone								
Jimma	55.8	49.7	52.2	50.0	55.7	49.7	54.5	49.8
West Hararghe	44.2	49.7	47.8	50.0	44.3	49.7	45.5	49.8
Average household size	5.3	0.4	5.6	0.4	5.0	0.2	5.2	0.1
Mean age of head of household	43.0	3.3	43.1	3.2	43.5	2.6	43.6	2.4
Average number of eligible women	1.0	0.1	1.0	0.1	1.0	0.0	1.0	0.0
Average number of eligible children	0.9	0.2	0.9	0.2	0.8	0.1	0.8	0.1
Percent female head of household	13.2	3.3	16.7	6.6	14.0	3.2	14.8	3.0
Wealth quintiles of household population	%		%		%		%	
First	23.1	27.8	20.2	24.9	23.3	20.7	23.5	20.9
Second	17.5	13.0	20.6	12.0	20.2	8.6	20.6	5.5
Third	17.0	8.3	21.7	9.5	18.3	5.6	21.1	5.1
Fourth	18.7	14.3	20.4	14.5	18.9	10.5	19.0	9.4
Fifth	23.8	22.1	17.0	16.5	19.3	17.4	15.9	10.7
Women's education	%		%		%		%	
No schooling	74.1	12.9	73.3	12.2	70.7	8.7	70.6	8.3
Primary	21.2	9.9	22.8	9.8	26.3	7.1	26.5	7.3
Secondary or higher	4.7	4.0	3.8	3.2	3.0	2.2	2.9	2.1
Average age (in years) of caretaker of children under five	29.9	1.8	29.8	1.4	29.6	0.8	29.7	1.0
Average age (in months) of children under five	29.9	2.5	29.5	1.9	29.4	1.1	29.8	1.4
Average age (in months) of children aged 6–23 months	13.8	1.0	14.3	1.0	13.6	0.8	13.8	0.6

SD = standard deviation. Averages and percentages are based on unweighted averages and percentages at woreda level. Intervention areas have 16 woredas and comparison areas have 15 woredas.

**Table 2:** Percentage of children aged 2–59 months with pneumonia, fever, and diarrhea for whom care was sought, by type of care provider, according to study group at baseline and endline

Indicator	Intervention				Comparison				*P* value of difference in differences
	Baseline	Endline	Difference	*P* value	Baseline	Endline	Difference	*P* value	
Careseeking from formal provider*									
Pneumonia	28.8	41.1	12.3	0.085	27.4	39.9	12.4	0.072	0.841
Fever	21.2	25.3	4.1	0.315	25.1	29.5	4.4	0.226	0.780
Diarrhea	21.2	21.7	0.5	0.881	15.2	23.7	8.4	0.048	0.285
At least one illness	23.4	26.0	2.6	0.442	23.6	29.5	5.9	0.059	0.771
Careseeking from HEW									
Pneumonia	5.1	11.5	6.4	0.021	4.3	9.7	5.4	0.119	0.441
Fever	3.9	8.5	4.6	0.020	2.5	6.4	3.9	0.009	0.713
Diarrhea	6.2	9.9	3.7	0.220	3.4	8.3	5.0	0.010	0.658
At least one illness	5.1	9.3	4.2	0.031	3.6	7.7	4.1	0.015	0.656
Careseeking from other public health facility									
Pneumonia	14.1	25.3	11.2	0.100	11.0	24.7	13.7	0.013	0.536
Fever	9.9	12.8	2.9	0.322	13.1	16.3	3.2	0.345	0.956
Diarrhea	7.7	10.2	2.4	0.367	7.1	11.1	4.0	0.055	0.800
At least one illness	10.4	13.3	2.8	0.307	11.4	15.7	4.3	0.088	0.913
Careseeking from private health facility									
Pneumonia	12.7	6.7	−6.0	0.146	13.8	9.7	−4.1	0.288	0.760
Fever	9.5	5.4	−4.1	0.136	10.7	8.4	−2.3	0.326	0.844
Diarrhea	8.2	2.6	−5.6	0.025	5.8	5.5	−0.3	0.913	0.121
At least one illness	10.1	4.8	−5.3	0.028	10.1	7.9	−2.1	0.322	0.364
Careseeking from informal sources									
Pneumonia	13.3	0.2	−13.1	0.003	7.3	4.1	−3.2	0.188	0.015
Fever	13.7	0.6	−13.1	0.000	10.1	2.9	−7.2	0.014	0.034
Diarrhea	10.3	2.5	−7.8	0.009	10.0	3.8	−6.3	0.031	0.243
At least one illness	14.2	1.2	−13.1	0.000	11.6	4.0	−7.7	0.008	0.024

HEW = health extension worker. Percentage based on woreda averages. Questions on careseeking required respondent to list all place where they have sought care.

* Formal care providers include public and private health facilities, including HEWs at health posts.

**Table 3:** Percentage of children aged 2–59 months with suspected pneumonia, fever, and diarrhea who were treated with the recommended drug, by type of treatment provider, according to study group at baseline and endline

Indicator	Intervention				Comparison				P value of difference in differences
	Baseline	Endline	Difference	P value	Baseline	Endline	Difference	P value	
Treatment with recommended drug									
Antibiotics for pneumonia	31.0	17.7	−13.4	0.030	26.8	24.3	−2.6	0.678	0.414
Antimalarial for fever	3.8	2.9	−0.8	0.514	4.6	4.8	0.3	0.837	0.389
ORS for diarrhea	15.0	17.7	2.7	0.460	9.0	17.2	8.2	0.001	0.296
Combined	19.5	12.8	−6.6	0.018	17.9	16.5	−1.4	0.558	0.313
Treatment with recommended drug within 24 hours of onset of illness									
Suspected pneumonia	15.4	5.6	−9.8	0.027	12.3	8.2	−4.1	0.115	0.389
Fever	1.1	1.8	0.7	0.384	1.8	1.3	−0.4	0.580	0.281
Treatment from HEW									
Antibiotics for pneumonia	2.1	5.2	3.1	0.170	1.1	1.0	−0.1	0.933	0.140
Antimalarial for fever	0.9	0.9	0.0	0.990	1.9	0.4	−1.5	0.058	0.158
ORS for diarrhea	5.3	8.3	2.9	0.300	1.7	5.2	3.5	0.003	0.373
Combined	3.4	5.4	2.0	0.174	2.5	2.9	0.5	0.572	0.593
Treatment from other public health facility									
Antibiotics for pneumonia	9.7	8.2	−1.5	0.682	7.9	15.7	7.8	0.071	0.195
Antimalarial for fever	0.5	1.2	0.7	0.133	1.0	3.1	2.1	0.040	0.573
ORS for diarrhea	3.6	6.8	3.2	0.108	4.5	6.7	2.3	0.188	0.382
Combined	5.0	5.1	0.1	0.957	6.0	8.0	2.1	0.211	0.626
Treatment from private health facility									
Antibiotics for pneumonia	8.5	2.2	−6.3	0.021	9.4	4.6	−4.8	0.085	0.217
Antimalarial for fever	1.0	0.1	−0.9	0.071	1.0	0.8	−0.2	0.753	0.131
ORS for diarrhea	3.2	1.0	−2.2	0.067	1.5	3.8	2.3	0.176	0.046
Combined	5.2	1.1	−4.1	0.001	4.9	3.8	−1.1	0.471	0.023
Treatment from informal sources									
Antibiotics for pneumonia	10.4	2.9	−7.5	0.037	7.5	3.1	−4.4	0.139	0.885
Antimalarial for fever	1.0	0.8	−0.3	0.668	0.4	0.5	0.1	0.762	0.550
ORS for diarrhea	1.9	1.6	−0.3	0.824	0.4	1.6	1.2	0.083	0.050
Combined	5.8	1.5	−4.3	0.029	4.2	1.8	−2.4	0.154	0.486

HEW = health extension worker; ORS = oral rehydration salts. Percentage based on woreda averages.

**Table 4:** Mortality rate among children aged 2–59 months, by study group at baseline and endline

Mortality rate	Baseline		Endline		Absolute difference (endline–baseline)		Percent relative difference
	Value*	95% CI†	Value*	95% CI†	Value	95% CI	
Intervention arm	49.0	37.8–60.3	42.8	34.6–51.1	−6.2	−14.3–1.9	−12.7
Comparison arm	45.0	36.5–53.5	40.9	34.6–47.2	−4.1	−13.9–5.8	−9.1
Difference	−4.1	−17.7–9.6	−1.9	−12.0–8.09	2.1	−14.1–18.4	

* Based on average of mortality among children aged 2–59 across 16 woredas in intervention areas and 15 woredas in comparison areas.

† Based on *t* test.
