# Supplementary material for: Effects of the integrated Community Case Management of Childhood Illness Strategy on Child Mortality in Ethiopia: A Cluster Randomized Trial
Source: Am J Trop Med Hyg. 2016 Mar 2;94(3):596–604. doi: 10.4269/ajtmh.15-0586 (PMC4775896; doi:10.4269/ajtmh.15-0586)
Supplement: Supplementary file 1 [file SD8.pdf]

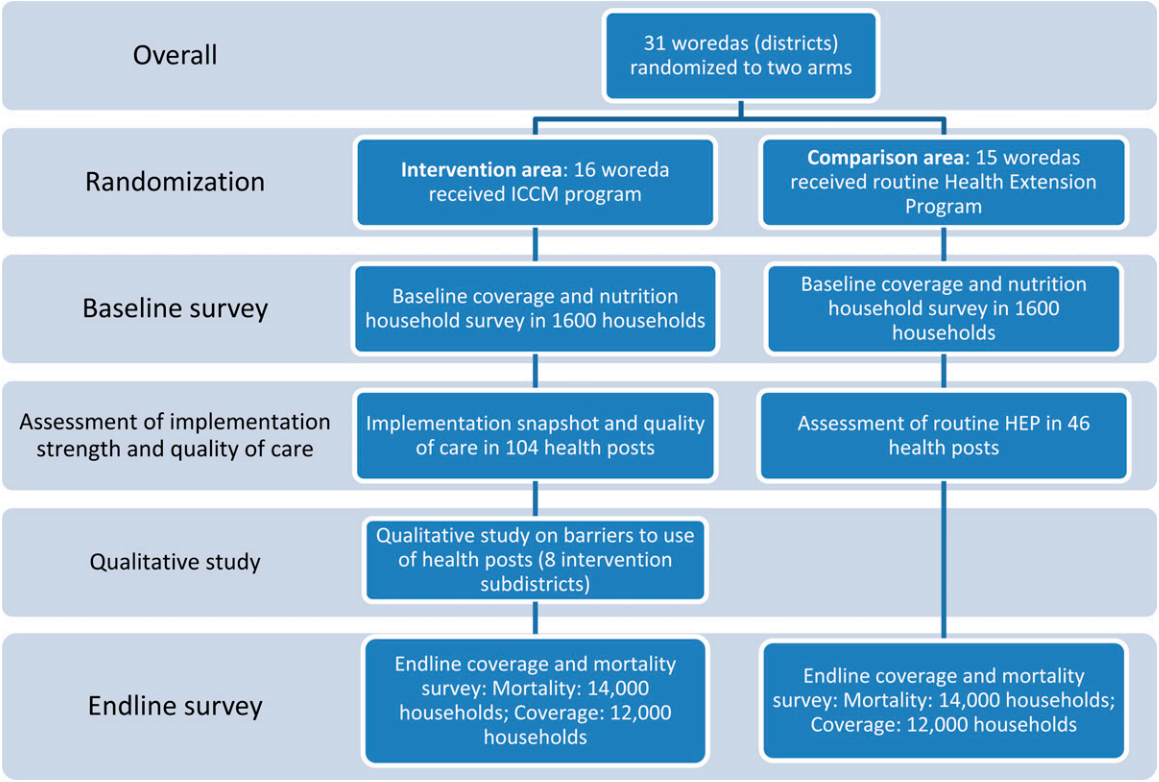

SUPPLEMENTAL FIGURE 1. Trial design.

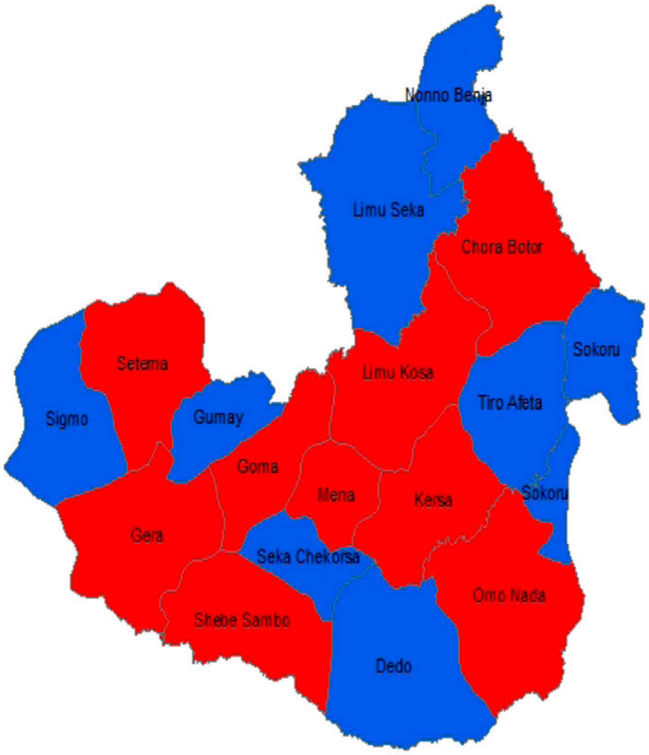

Note: Blue = Comparison, Red = Intervention

SUPPLEMENTAL FIGURE 2. Intervention and comparison woredas in Jimma zone.

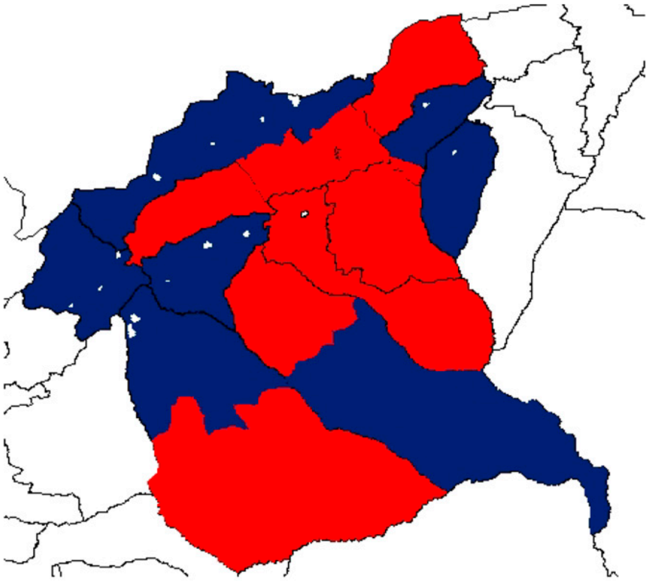

Note: Blue = Comparison, Red = Intervention

SUPPLEMENTAL FIGURE 3. Intervention and comparison woredas in West Hararghe zone.

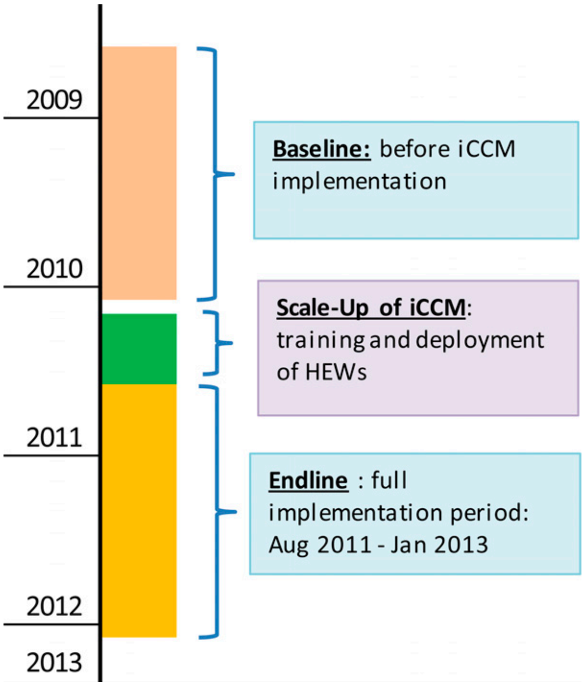

SUPPLEMENTAL FIGURE 4. Measurement period for mortality.

SUPPLEMENTAL TABLE 1

Comparison between intervention and comparison areas in terms of case management guidelines and program inputs for the Ethiopia iCCM program vs. routine HEP

Management of iCCM illnesses for children 2–59 months	ICCM (intervention areas)	Routine HEP (comparison areas)
Pneumonia	Antibiotics (cotrimoxazole)	Referral to health center
Severe pneumonia	Prereferral treatment with antibiotics<br>Referral to health center	Referral to health center
Diarrhea (some dehydration, no dehydration)	ORS/ORT<br>Zinc	ORS/ORT
Severe diarrhea (severe dehydration, persistent diarrhea, severe persistent diarrhea, dysentery)	ORS<br>Vitamin A (for persistent and severe persistent diarrhea only)<br>Referral to health center	ORS<br>Vitamin A (for persistent and severe persistent diarrhea only)<br>Referral to health center
Malaria	Antimalarial (ACT after positive RDT)	Antimalarial (ACT after positive RDT)
Severe febrile disease	Pre-referral treatment with antibiotics<br>Referral to health center	Referral to health center
Uncomplicated malnutrition	Ready to use therapeutic food (RUTF) or supplementary feeding program	RUTF or supplementary feeding program
Severe complicated malnutrition	Pre-referral treatment with amoxicillin and vitamin A<br>Referral to health center	Pre-referral treatment with amoxicillin and vitamin A<br>Referral to health center
Measles	Vitamin A	Vitamin A
Severe complicated measles	Vitamin A<br>Antibiotics (cotrimoxazole)<br>Tetracycline eye ointment (optional)<br>Referral to health center	Vitamin A<br>Antibiotics (cotrimoxazole)<br>Tetracycline eye ointment (optional)<br>Referral to health center
Measles with eye or mouth complications	Vitamin A<br>Tetracycline eye ointment (optional)<br>Gentian violet (optional)	Vitamin A<br>Tetracycline eye ointment (optional)<br>Gentian violet (optional)
Acute ear infection	Paracetamol<br>Referral to health center	Paracetamol<br>Referral to health center
Anemia	Referral to health center	Referral to health center
Program inputs		
Training of HEWs	6-day training on iCCM	No additional training
Supervision	Standardized supportive supervision on iCCM supported by partner NGOs plus standard government supervision<br>Biannual PRCM meetings	Standard government supervision
Supply of commodities	Support for purchase and supply of drugs and other commodities by UNICEF and partners<br>Provision of iCCM registers, iCCM chart booklets, timers, and other supplies	Standard government commodity supply chain system<br>No additional supplies or job aids
Monitoring and evaluation	Enhanced data collection during supervisions and PRCM meetings<br>Data management support by UNICEF	Standard government monitoring and evaluation

ACT = artemisinin-based combination therapy; HEP = Health Extension Program; iCCM = integrated community case management; ORS = oral rehydration salts; ORT = oral rehydration therapy; PRCM = performance review and clinical mentoring; RDT = rapid diagnostic test; RTUF = ready-to-use therapeutic foods; UNICEF = United Nations Children's Fund.

Supplemental Table 2

Sample size calculation for the endline survey

Line no.	U5MR		
1	Year 2000	0.135	DHS 2005 (10-year rate)
2	Year 2005	0.114	DHS 2010 (10-year rate)
3	Annual rate of change	−3.32%	
4	Corresponding rate of change for 18 months	−4.93%	Mortality rate at 18 months assumed equal to average rates at 12 and 24 months (using formula 0.5 (r + 1) (r + 2) − 1, where r is the annual rate of change)
5	Rural U5MR in 2005	0.114	From DHS 2005 (10-year mortality rate)
6	Rural U5MR in 2010	0.096	Estimated assuming annual rate of change in line 3
7	Total number of households		
8	Intervention	556,366	
9	Control	483,893	
10	Baseline U5MR		
11	Phase I area	0.096	From line 6 above
12	Phase II area	0.096	
13	Endline U5MR		
14	Phase I area (intervention areas)	71.303	Assumed 21% decline above the secular trends (−25.93%)
15	Phase II area (comparison areas)	91.519	Assumed secular trends at national rate of change (−4.93%)
16	Assumptions		
17	Power	0.8	
18	Alpha	0.05	
19	Design effect	1.2	From DHS 2010 (Oromia region)
20	Crude birth rate	0.036	
21	Household size	5	
22	Non response rate	0.05	
23	Two sided test; conservative approach assuming independent samples		
24	Sample size of births	2,969	Using STATA sampsi command comparing intervention and comparison at endline
25	Corresponding number of households	13,890	
26	Total sample of households in the two arms	27,780	Rounded to 28,000 households

DHS = demographic and health surveys.

Supplemental Table 3

Percentage of children under five and children aged 2–59 months who had pneumonia, fever, or diarrhea in the 2 weeks preceding the baseline and endline surveys and percentage for whom care was sought from a formal provider, by study group

	Intervention				Comparison				
Indicator	Baseline	Endline	Difference	*P* value	Baseline	Endline	Difference	*P* value	*P* value of difference in differences
Careseeking from formal provider*									
Children aged 0–59 months									
Pneumonia	28.3	39.4	11.0	0.1178	27.2	39.6	12.4	0.0715	0.8630
Fever	20.9	25.1	4.2	0.3043	24.9	29.4	4.5	0.2089	0.7960
Diarrhea	20.8	21.5	0.7	0.8188	15.3	23.6	8.3	0.0478	0.3130
At least one illness	22.9	25.7	2.8	0.4233	23.3	29.3	5.9	0.0545	0.7680
Children aged 2–59 months									
Pneumonia	28.8	41.1	12.3	0.0850	27.4	39.9	12.4	0.0722	0.8410
Fever	21.2	25.3	4.1	0.3147	25.1	29.5	4.4	0.2257	0.7800
Diarrhea	21.2	21.7	0.5	0.8807	15.2	23.7	8.4	0.0481	0.2850
At least one illness	23.4	26.0	2.6	0.4417	23.6	29.5	5.9	0.0594	0.7710

*Formal care providers include public and private health facilities, including health posts.

Supplemental Table 4

Percentage of children, by study group at baseline and endline, under five and children aged 2–59 months with pneumonia, fever, and diarrhea for whom care was sought, by type of care provider

Indicator	Intervention				Comparison				*P* value of difference in differences
	Baseline	Endline	Difference	*P* value	Baseline	Endline	Difference	*P* value	
Careseeking to HEW									
Children aged 0–59 months									
Pneumonia	4.9	11.5	6.6	0.0174	4.3	9.6	5.4	0.1208	0.4560
Fever	3.8	8.4	4.7	0.0169	2.4	6.3	3.9	0.0088	0.7120
Diarrhea	6.1	9.8	3.7	0.2074	3.3	8.5	5.1	0.0090	0.6240
At least one illness	5.0	9.2	4.3	0.0256	3.5	7.7	4.2	0.0146	0.6430
Children aged 2–59 months									
Pneumonia	5.1	11.5	6.4	0.0211	4.3	9.7	5.4	0.1185	0.4410
Fever	3.9	8.5	4.6	0.0197	2.5	6.4	3.9	0.0086	0.7130
Diarrhea	6.2	9.9	3.7	0.2200	3.4	8.3	5.0	0.0098	0.6580
At least one illness	5.1	9.3	4.2	0.0313	3.6	7.7	4.1	0.0149	0.6560
Careseeking to other public health facility									
Children aged 0–59 months									
Pneumonia	13.8	23.6	9.8	0.1252	11.0	24.5	13.5	0.0145	0.5300
Fever	9.7	12.7	3.0	0.2888	13.0	16.3	3.3	0.3311	0.9540
Diarrhea	7.5	10.1	2.6	0.3310	7.2	11.0	3.7	0.0670	0.8900
At least one illness	10.2	13.1	2.9	0.2907	11.3	15.6	4.3	0.0896	0.9220
Children aged 2–59 months									
Pneumonia	14.1	25.3	11.2	0.1000	11.0	24.7	13.7	0.0132	0.5360
Fever	9.9	12.8	2.9	0.3216	13.1	16.3	3.2	0.3454	0.9560
Diarrhea	7.7	10.2	2.4	0.3674	7.1	11.1	4.0	0.0550	0.8000
At least one illness	10.4	13.3	2.8	0.3071	11.4	15.7	4.3	0.0882	0.9130
Careseeking to private health facility									
Children aged 0–59 months									
Pneumonia	12.6	6.5	−6.2	0.1326	13.6	9.7	−3.9	0.3081	0.7470
Fever	9.5	5.3	−4.2	0.1415	10.6	8.3	−2.3	0.3426	0.8120
Diarrhea	8.1	2.6	−5.5	0.0264	5.7	5.4	−0.3	0.9169	0.1250
At least one illness	10.1	4.8	−5.3	0.0308	9.9	7.8	−2.1	0.3160	0.3670
Children aged 2–59 months									
Pneumonia	12.7	6.7	−6.0	0.1463	13.8	9.7	−4.1	0.2875	0.7600
Fever	9.5	5.4	−4.1	0.1361	10.7	8.4	−2.3	0.3258	0.8440
Diarrhea	8.2	2.6	−5.6	0.0247	5.8	5.5	−0.3	0.9126	0.1210
At least one illness	10.1	4.8	−5.3	0.0283	10.1	7.9	−2.1	0.3217	0.3640
Careseeking from informal sources									
Children aged 0–59 months									
Pneumonia	12.7	0.2	−12.5	0.0020	7.0	4.1	−2.9	0.2083	0.0150
Fever	13.9	0.6	−13.3	0.0003	9.8	2.9	−6.9	0.0146	0.0300
Diarrhea	10.3	2.5	−7.8	0.0095	9.8	3.7	−6.1	0.0323	0.2430
At least one illness	14.3	1.2	−13.1	0.0081	11.3	3.9	−7.4	0.0002	0.0220
Children aged 2–59 months									
Pneumonia	13.3	0.2	−13.1	0.0026	7.3	4.1	−3.2	0.1883	0.0150
Fever	13.7	0.6	−13.1	0.0004	10.1	2.9	−7.2	0.0137	0.0340
Diarrhea	10.3	2.5	−7.8	0.0094	10.0	3.8	−6.3	0.0306	0.2430
At least one illness	14.2	1.2	−13.1	0.0003	11.6	4.0	−7.7	0.0076	0.0240

HEW = health extension worker. Percentage based on woreda averages.

Supplemental Table 5

Percentage of children under five and children aged 2–59 months with pneumonia, fever, and diarrhea in the 2 weeks preceding the baseline and endline surveys who received the recommended drug treatment, by type of illness and study group

Indicator	Intervention				Comparison				*P* value of difference in differences
	Baseline	Endline	Difference	*P* value	Baseline	Endline	Difference	*P* value	
Treatment with recommended drug									
Children aged 0–59 months									
Antibiotics for pneumonia	30.5	17.2	−13.3	0.0267	26.1	24.1	−2.0	0.7366	0.4070
Antimalarial for fever	3.7	2.9	−0.8	0.5384	4.5	4.8	0.3	0.8271	0.3870
ORS for diarrhea	14.7	17.6	2.9	0.4288	9.1	17.2	8.1	0.0012	0.3350
ORS + zinc for diarrhea	0.4	1.0	0.6	0.4761	0.0	0.6	0.6	0.3259	N/A
Combined	18.9	12.7	−6.2	0.0228	17.6	16.4	−1.1	0.6307	0.3210
Children aged 2–59 months									
Antibiotics for pneumonia	31.0	17.7	−13.4	0.0296	26.8	24.3	−2.6	0.6783	0.4140
Antimalarial for fever	3.8	2.9	−0.8	0.5139	4.6	4.8	0.3	0.8373	0.3890
ORS for diarrhea	15.0	17.7	2.7	0.4597	9.0	17.2	8.2	0.0012	0.2960
ORS + zinc for diarrhea	0.4	1.0	0.6	0.4761	0.0	0.6	0.6	0.3259	N/A
Combined	19.5	12.8	−6.6	0.0183	17.9	16.5	−1.4	0.5579	0.3130
Treatment with recommended drug within 24 hours of onset of illness									
Children 0–59 months									
Pneumonia	15.3	5.6	−9.7	0.0295	11.9	8.0	−3.9	0.1402	0.4190
Fever	1.0	1.7	0.7	0.3436	1.7	1.3	−0.4	0.5736	0.2830
Children 2–59 months									
Pneumonia	15.4	5.6	−9.8	0.0267	12.3	8.2	−4.1	0.1145	0.3890
Fever	1.1	1.8	0.7	0.3844	1.8	1.3	−0.4	0.5799	0.2810

ORS = oral rehydration salts. Percentage based on woreda averages; N/A = difference in differences model did not converge.

Supplemental Table 6

Percentage of children under five and aged 2–59 months with pneumonia, fever, and diarrhea who were treated with the recommended drug, by type of treatment provider at baseline and endline, by study group

Indicator	Intervention				Comparison				*P* value of difference in differences
	Baseline	Endline	Difference	*P* value	Baseline	Endline	Difference	*P* value	
Treatment from HEW									
Children aged 0–59 months									
Antibiotics for pneumonia	2.1	5.2	3.1	0.1666	1.1	1.0	−0.1	0.9569	0.1420
Antimalarial for fever	0.8	0.9	0.0	0.9562	1.9	0.4	−1.5	0.0581	0.1590
ORS for diarrhea	5.2	8.2	2.9	0.2964	1.6	5.3	3.7	0.0026	0.3400
Combined	3.3	5.4	2.1	0.1509	2.4	3.0	0.6	0.4761	0.6360
Children aged 2–59 months									
Antibiotics for pneumonia	2.1	5.2	3.1	0.1698	1.1	1.0	−0.1	0.9328	0.1400
Antimalarial for fever	0.9	0.9	0.0	0.9902	1.9	0.4	−1.5	0.0581	0.1580
ORS for diarrhea	5.3	8.3	2.9	0.2999	1.7	5.2	3.5	0.0026	0.3730
Combined	3.4	5.4	2.0	0.1738	2.5	2.9	0.5	0.5723	0.5930
Treatment from other public health facility									
Children aged 0–59 months									
Antibiotics for pneumonia	9.5	7.8	−1.7	0.5948	8.1	15.3	7.2	0.0752	0.2020
Antimalarial for fever	0.5	1.2	0.7	0.1344	1.0	3.1	2.1	0.0397	0.5700
ORS for diarrhea	3.5	6.7	3.2	0.0982	4.4	6.6	2.2	0.1896	0.3740
Combined	4.8	5.0	0.2	0.8947	6.0	7.9	1.9	0.2278	0.6790
Children aged 2–59 months									
Antibiotics for pneumonia	9.7	8.2	−1.5	0.6823	7.9	15.7	7.8	0.0708	0.1950
Antimalarial for fever	0.5	1.2	0.7	0.1332	1.0	3.1	2.1	0.0404	0.5730
ORS for diarrhea	3.6	6.8	3.2	0.1083	4.5	6.7	2.3	0.1875	0.3820
Combined	5.0	5.1	0.1	0.9569	6.0	8.0	2.1	0.2105	0.6260
Treatment from private health facility									
Children aged 0–59 months									
Antibiotics for pneumonia	8.4	2.0	−6.4	0.0146	9.2	4.6	−4.6	0.0955	0.2130
Antimalarial for fever	0.9	0.1	−0.8	0.0709	1.0	0.8	−0.2	0.7446	0.1300
ORS for diarrhea	3.1	1.0	−2.1	0.0710	1.5	3.8	2.3	0.1798	0.0480
Combined	5.0	1.0	−4	0.0000	4.8	3.8	−1	0.4834	0.0250
Children aged 2–59 months									
Antibiotics for pneumonia	8.5	2.2	−6.3	0.0208	9.4	4.6	−4.8	0.0854	0.2170
Antimalarial for fever	1.0	0.1	−0.9	0.0711	1.0	0.8	−0.2	0.7529	0.1310
ORS for diarrhea	3.2	1.0	−2.2	0.0670	1.5	3.8	2.3	0.1758	0.0460
Combined	5.2	1.1	−4.1	0.0005	4.9	3.8	−1.1	0.4709	0.0230
Treatment from informal sources									
Children aged 0–59 months									
Antibiotics for pneumonia	10.1	2.9	−7.2	0.0404	7.2	3.0	−4.2	0.1399	0.8810
Antimalarial for fever	1.0	0.7	−0.3	0.6820	0.4	0.5	0.1	0.7428	0.5470
ORS for diarrhea	1.9	1.6	−0.3	0.8293	0.7	1.6	1.0	0.2151	0.1030
Combined	5.7	1.5	−4.2	0.0309	4.2	1.8	−2.4	0.1424	0.5190
Children aged 2–59 months									
Antibiotics for pneumonia	10.4	2.9	−7.5	0.0369	7.5	3.1	−4.4	0.1394	0.8850
Antimalarial for fever	1.0	0.8	−0.3	0.6682	0.4	0.5	0.1	0.7622	0.5500
ORS for diarrhea	1.9	1.6	−0.3	0.8239	0.4	1.6	1.2	0.0826	0.0500
Combined	5.8	1.5	−4.3	0.0290	4.2	1.8	−2.4	0.1539	0.4860

ORS = oral rehydration salts.

Supplemental Table 7

Mortality rate among children under five and children aged 2–59 months by evaluation group

Mortality rate	Baseline				Endline				Absolute difference (endline − baseline)		Percent relative difference
	Value*	SE†	95% CI†	Range‡	Value*	SE†	95% CI†	Range‡	Value	95% CI	
Under-five mortality rate											
Intervention group	81.0	5.4	69.4–92.5	49.9–114.8	78.5	5.1	67.7–89.3	(53.4–116.8)	−2.5	−14.7–9.8	−3.0
Comparison group	76.7	6.6	62.6–90.8	35.6–109.8	70.3	5.5	58.6–82.0	(44.1–109.8)	−6.4	−23.6–10.8	−8.3
Difference (comparison intervention)	−4.3	8.5	−21.6–13.1	–	−8.2	7.4	23.4–7.0	–	**−3.9**	**−26.0–12.9**	–
Mortality among children 2–59 months											
Intervention group	49.0	5.3	37.8–60.3	22.8–90.5	42.8	3.9	34.6–51.1	(19.0–76.9)	−6.2	−14.3–1.9	−12.7
Comparison group	45.0	4.0	36.5–53.5	12.4–64.9	40.9	2.9	34.6–47.2	(25.6–64.9)	−4.1	−13.9–5.8	−9.1
Difference (comparison intervention)	−4.1	6.7	−17.7–9.6	–	−1.9	4.9	−12.0–8.09	–	**2.1**	**−14.1–18.4**	–

95% CI = 95% confidence interval; SE = standard error. Bold values are not statistically significant, judging by the 95% confidence intervals that do not include 0.
*Based on average of U5MR across woredas.
†Based on *t* test.
‡Across 16 woredas in intervention areas and 15 woredas in comparison areas.

Supplemental Table 8

Changes in selected other indicators

Indicator	Intervention				Comparison				P value of difference in differences†
	Baseline	Endline	Difference	P value*	Baseline	Endline	Difference	P value*	
ITN use by children under five	12.7	10.6	−2.1	0.5357	17.8	7.7	−10.1	0.0356	0.3240
Exclusive breast-feeding (0–6 months)	33.8	60.1	26.3	0.0001	42.8	56.3	13.5	0.0169	0.1130
Immunization (12–23 months)									
Measles (1+)	64.4	38.1	−26.3	0.0000	72.3	37.7	−34.7	0.0000	0.2520
Pentavalent (3+)	27.7	27.4	−0.2	0.9608	26.8	25.7	−1.1	0.8212	0.9330
Pneumococcal (3+)	0.0	20.2	20.2	0.0000	0.0	17.2	17.2	0.0000	0.9980
Vitamin A supplementation in previous 12 months (6–59 months)	71.7	70.3	−1.4	0.7556	80.0	65.9	−14.2	0.0025	0.0030

Percentage based on woreda averages. ITN = insecticide treated net.
*Based on *t* test across woreda averages.
†Based on Generalized mixed model with logit link.
